# Claire Wardle: tackling the infodemic

**DOI:** 10.2471/BLT.21.030321

**Published:** 2021-03-01

## Abstract

Claire Wardle talks to Andréia Azevedo Soares about the challenges faced in addressing false or misleading information about health.

Q: How did you come to focus on the issue of misinformation?

A: I became interested in user-generated Internet content as an academic and ended up doing a big piece of research for the BBC (British Broadcasting Corporation) in 2008. That research led to the BBC inviting me to spend six months working with their newsrooms to implement some of the recommendations I’d made about vetting user-generated content. After the first two weeks, I felt like I’d had more valuable conversations in BBC elevators than I'd had in the previous five years in academia. I also realized that I had been writing journal articles that nobody in the newsrooms would read, even though my focus was how to improve the media industry. Moving out of academia was a big step for me, but I never looked back. I co-founded First Draft in 2015, and now work full-time training journalists in information verification best practice, conducting research in the information landscape and helping international newsrooms spot and debunk misinformation.

Q: How did First Draft come into being?

A: We were founded by nine organizations brought together by the Google News Lab, which is the part of Google that works with news organizations to help drive innovation and provide training on and access to emerging technologies. We were initially focused on web-based training of journalists in how to verify social media content, especially eye-witness content generated during breaking news events, and how to verify content and identify sources. Then in 2017, partly in response to interest in the role played by social media in the US presidential election, our focus shifted to the arena of misinformation, which we define as the inadvertent sharing of false information, and disinformation, the term we use for the deliberate creation and sharing of information known to be false.

Q: How does First Draft engage with these different issues?

A: First Draft’s activities break down into three core functions: projects in the field, research and media training. Each plays an important role in achieving our main aim which is to support the identification and exposure of misinformation. Our first field project, called Crosscheck, was implemented in France in 2017 and brought together 37 French newsrooms to help them identify and report false, misleading and confusing content that circulated in the weeks leading up to the French presidential election. All the newsrooms worked together, cross-checking each other’s stories, and also putting their logos side by side when validating verified content to boost trust among their consumers. The project demonstrated how collaborative journalism not only helps build trust but can also support verification capacity-building. Since then we have implemented Crosscheck projects in several countries, showing how competing newsrooms can work together to generate more effective, efficient and responsible reporting. We also monitor content ourselves using a team of 20 journalists distributed worldwide and using content discovery and analytics tools that report on what’s happening across social media and how content is being shared.

“The more that uncertainty […] is made public, the more it is weaponized by bad actors.”

Q: Do you make use of artificial intelligence (AI) or machine learning (ML) to support your monitoring?

A: AI and, to a much lesser degree, ML are built into the tools we are using, and I think over the next five to 10 years AI will help us speed up and automate some of our activities, but I don't think it will ever entirely replace human beings. This is partly because many of the players generating misinformation are using techniques designed to fool AI: for example, by using genuine content but then reframing or tweaking it to get a different message across. In fact, most effective disinformation has a kernel of truth to it. AI tends to work quite well with graphic content and nudity, but it is not so good at detecting or interpreting the kind of satire or irony that you might find in an Instagram meme. So human eyes and brains are still required to identify and interpret it.

Q: To what extent do you monitor content in the public health arena?

A: We have a team that focuses solely on health and science, drawing on the lessons we have learned over five years monitoring political misinformation. The team follows targeted accounts, monitoring hashtag changes which can be used to evade bans, and examining narratives to ensure that false or misleading narratives are identified, exposed and debunked. We also have a big network of journalists around the world and communicate with them on an online platform which we use to put out alerts, do briefings and share documents daily.

Q: Can you give examples of your work in relation to COVID-19?

A: At the beginning of the COVID-19 outbreak, we monitored and subsequently shared briefing documents about the hashtag #filmyourhospital, which became popular on Twitter as part of a movement to ‘prove’ that the pandemic was a hoax by showing ‘empty’ hospitals. We did similar work on hydroxychloroquine regarding narratives supporting claims of its efficacy in ‘treating’ COVID-19. We also conducted research over the summer of 2020 regarding narratives about vaccines.

Q: Do you work directly with the public health community?

A: We are beginning to and our goal in 2021 is to work more directly with public health professionals. Personally, I would love to be able to get them involved and am very encouraged by the efforts the World Health Organization (WHO) has made in this area. A good example is the infodemic management training webinar that WHO launched in June 2020. The webinar brought together medical researchers, social scientists, journalists and health professionals to talk about the ways in which misinformation impacts their ability to deliver timely, relevant information. 

Q: What are the challenges faced in ensuring optimal dissemination of information about COVID-19?

A: The fact that the information itself is constantly evolving in response to the developing evidence base and the developing virus is one. This has sometimes given the impression that the authorities themselves don’t ‘know’ what they are talking about. There have been several instances of this. For example, regarding messaging around masks, airborne transmission of the virus, and vaccine dosage. Experts were actually using Twitter to publicly discuss whether a given vaccine required one or two doses. Such conversations should probably not happen in the public space in real time. Similarly, daily briefings on the pandemic may not be the optimal conduit for the dissemination of information. Generally speaking, the more that uncertainty, especially uncertainty about risk, is made public, the more it is weaponized by bad actors in the information sphere. Communicating complexity is another obvious challenge. This is acknowledged in WHO’s definition of an infodemic which includes the notion of an overabundance of information, whether accurate or not. Too much information makes it harder for people to identify core messages and reliable guidance from trusted sources when they need it. The communication of complexity is something that public health organizations have often struggled with, partly because they have tended to adopt a paternalistic science-based approach. They also typically broadcast information as though social media represented one homogenous space.

“It would be difficult to overstate the challenges faced.”

*Q: Can you say more about that?*

A: The public broadcasting approach may have made sense 30 years ago when there were just a few sources or gatekeepers who everyone relied on, but the information landscape has changed and become much more fragmented. These days many people gather or absorb their information through trusted social influencers rather than ‘official outlets’. This has important implications for the way public health information should be communicated. There has also been a tendency to over-rely on the self-evident nature of scientific content without giving sufficient consideration to the way people process information and messages through the filter of their beliefs, biases and emotions.

Q: Are you suggesting that public health messaging should be simpler or more emotional?

A: No, but public health communicators need to find more engaging ways of getting their message across that allows for an emotional connection. There have been one or two good examples of this. For example, a number of countries adopted the ‘flatten the curve’ messaging as a part of efforts to encourage social distancing and other measures. The flatten the curve meme is generally linked to messages about mitigating the strain on health systems and more specifically the physical and mental strain on individual health workers. It is a perfect illustration of how to get across a fairly abstract concept in an easy-to-understand way, while also making an appeal to people’s sense of solidarity with the doctors and nurses on the front line. This kind of emotionalization is at the core of effective communication and is often used by misinformation agents who use it to drive their stories home.

Finally, given their relative lack of resources, it is important to recognize the limited capacity of public health communicators to make themselves heard. It is therefore essential that they engage with other stakeholders. This is already happening, with organizations such as WHO and the US Centers for Disease Control and Prevention establishing alliances with tech companies, several of which have implemented changes designed to direct attention towards authoritative content. A good example of this is WHO’s launching of a Facebook Messenger version of its WHO Health Alert platform – offering instant and accurate information about COVID-19. In other examples Google added a COVID-19 portal, and Twitter has significantly strengthened its policies regarding content that goes against guidance from authoritative sources of public health information. Whether or not such initiatives will be enough to make a difference going forward remains to be seen. 

